# Oxygen uptake on-kinetics during horizontal and uphill treadmill running in moderate- and severe-intensity domains

**DOI:** 10.1007/s00421-026-06292-9

**Published:** 2026-06-13

**Authors:** Thiago Pereira Ventura, Fernando Klitzke Borszcz, Gelson Ribeiro da Silva, Lucas Dalla Vecchia Lanzarini, Emmanuel de Assis Antunes de Farias, Ricardo Dantas de Lucas, Tiago Turnes

**Affiliations:** 1 Physical Effort Laboratory, CDS, UFSC, Sports Center, Federal University of Santa Catarina, Trindade, Florianópolis, SC CEP 88040900 Brazil; 2 Human Performance Research Group, Center for Health and Sport Sciences, Santa Catarina State University, Florianópolis, SC Brazil; 3 Department of Animal and Food Production, Agroveterinary Sciences Center, Santa Catarina State University, Lages, SC Brazil

**Keywords:** Trail running, Oxygen uptake kinetics, Exercise intensity domains

## Abstract

**Purpose:**

To compare pulmonary oxygen uptake (${\rm{\dot V}}{{\rm{O}}_{\rm{2}}}$) on-kinetics during level and uphill treadmill running performed within moderate- and severe-intensity domains in trained trail runners.

**Methods:**

Thirteen trail runners completed: (I) a traditional 3 min stage treadmill incremental test and (II) an incremental test at a fixed treadmill speed (50% of participant’s peak speed from the first test) with a 2% gradient increase every 3 min to determine the first lactate threshold (LT_1_) and ${\rm{\dot V}}{{\rm{O}}_{{\rm{2max}}}}$. On separate days, participants performed three 6-min square-wave transitions at 90% LT_1_ (moderate domain) and one 6-min transition at 70%Δ (severe domain) under both level and uphill conditions. Pulmonary ${\rm{\dot V}}{{\rm{O}}_{\rm{2}}}$ was measured breath-by-breath to determine primary amplitude, time delay, time constant (τp), and slow component (${\rm{\dot V}}{{\rm{O}}_{\rm{2SC}}}$), as well as blood lactate concentration ([La]) pre- and post-exercise.

**Results:**

During moderate exercise, τp and time delay did not differ between level and uphill running, whereas the primary ${\rm{\dot V}}{{\rm{O}}_{\rm{2}}}$ amplitude and post-exercise [La] were higher during uphill running (*p* < 0.05). In the severe domain, no differences were observed between conditions for τp, time delay, primary amplitude, or ${\rm{\dot V}}{{\rm{O}}_{\rm{2SC}}}$ amplitude. Peak ${\rm{\dot V}}{{\rm{O}}_{\rm{2}}}$ during severe exercise was higher in uphill compared with level running (*p* < 0.05), but values were similar to those obtained during the respective incremental tests.

**Conclusions:**

When exercise intensity domains are defined using slope-specific incremental testing, ${\rm{\dot V}}{{\rm{O}}_{\rm{2}}}$ on-kinetics are similar between level and uphill running in trail runners. These findings support the validity of incline-based testing for physiological normalization and training prescription in uphill running contexts.

## Introduction

Running intensity domains are defined based on distinct physiological responses and are commonly classified as: (a) moderate, which encompasses intensities at or below the first lactate threshold (LT_1_); (b) heavy, which includes intensities above LT_1_, but below the maximal lactate steady state or critical speed; (c) severe, which comprises exercise intensities that lead to maximal oxygen uptake (${\rm{\dot V}}{{\rm{O}}_{{\rm{2max}}}}$); and (d) extreme, defined by intensities at which the end of exercise precedes the attainment of ${\rm{\dot V}}{{\rm{O}}_{{\rm{2max}}}}$ (Burnley and Jones 2007; Turnes et al. 2016; Coates et al. 2023).

After the onset of moderate-intensity exercise, pulmonary O_2_ uptake (${\rm{\dot V}}{{\rm{O}}_{{\rm{2}}}}$) rises with an exponential kinetics to achieve a steady state within 2–3 min in healthy adults (Poole and Jones 2012; de Aguiar et al. 2022). In this regard, the primary time constant (tau – τ_p_) describing the rate at which ${\rm{\dot V}}{{\rm{O}}_{{\rm{2}}}}$ exponentially rises in the fundamental phase (phase II) to attain a steady state may represent an important determinant of O_2_ delivery and utilization for the exercising muscles (Poole and Jones 2012; de Aguiar et al. 2022), with important implications for exercise tolerance (Turnes et al. 2016; Murgatroyd et al. 2011). In the heavy- and severe-intensity domains, phase II is supplemented by a delayed-onset ${\rm{\dot V}}{{\rm{O}}_{{\rm{2}}}}$ slow component (V̇O_2SC_), which delays the attainment of the predictable ${\rm{\dot V}}{{\rm{O}}_{{\rm{2}}}}$ steady state (Burnley and Jones 2007; Jones et al. 2010; Turnes et al. 2016). This phenomenon has been related to increased metabolic inefficiency within the active muscles and is partly associated with additional motor unit recruitment (Krustrup et al. 2004; Jones et al. 2010; Grassi et al. 2015; Tam et al. 2024), although some studies suggest that this additional ${\rm{\dot V}}{{\rm{O}}_{{\rm{2}}}}$ (i.e. ${\rm{\dot V}}{{\rm{O}}_{{\rm{2SC}}}}$) could be attributed to the slow kinetics of type II muscle fiber that were recruited at the beginning of exercise (de Lima et al. 2020).

As ${\rm{\dot V}}{{\rm{O}}_{{\rm{2}}}}$ on-kinetics determine aerobic energy provision during exercise, understanding the mechanisms by which ${\rm{\dot V}}{{\rm{O}}_{{\rm{2}}}}$ on-kinetics may be impacted by exercise mode is relevant for different athletic populations (Rossiter 2011; Poole and Jones 2012; Tam et al. 2024). Interestingly, off-road running has increased in popularity worldwide in recent decades (Scheer et al. 2020). As it involves multiple uphill and downhill sections, this type of running practice challenges our current understating of physiological and biomechanical factors, which are typically assessed in flat conditions (Vernillo et al. 2017). In this regard, little is known about the potential differences in V̇O_2_ on-kinetics responses between level and uphill running in trail runners. Therefore, the manipulation of the treadmill slope during running may better represent the physiological and biomechanical specificity of uphill running.

Uphill running elicits high concentric muscle forces and/or prolonged stride duration imposed by slower speeds (Padulo et al. 2012), modifying kinematics parameters such as step frequency (SF) and step length (SL), and increasing the energy cost for a given speed (Looney et al. 2025). Additionally, increasing the treadmill slope changes the ratio of concentric to eccentric external work from 1:1 at a 0% gradient to 9:1 at a 10% gradient (Minetti et al. 1994). Furthermore, concentric muscle contraction preceded by eccentric muscle action produces a higher efficiency than isolated concentric contractions (Aura and Komi 1986; Ryschon et al. 1997), due to the recovery of elastic energy during the stretch-shortening cycle, which reduces the energy cost of locomotion and may help preserve muscle force (de Haan et al. 1991).

In this context, the pattern or regime of muscle contraction may play a role in modulating motor unit recruitment and the development of the ${\rm{\dot V}}{{\rm{O}}_{{\rm{2SC}}}}$ (Carter et al. 2000; Jones and McConnell 1999). However, few studies have investigated the influence of uphill running on ${\rm{\dot V}}{{\rm{O}}_{{\rm{2}}}}$ on-kinetics (Pringle et al. 2002; Willis et al. 2019). During moderate and heavy exercise, Pringle et al. (2002) observed similar phase II kinetics in healthy individuals, while uphill running on a 10% grade resulted in higher ${\rm{\dot V}}{{\rm{O}}_{\rm{2}}}{\rm{peak}}$ and ${\rm{\dot V}}{{\rm{O}}_{{\rm{2SC}}}}$ amplitude during heavy exercise. However, the method employed by the authors to determine exercise intensity, 50% of the difference between ${\rm{\dot V}}{{\rm{O}}_{{\rm{2}}}}$ at the first ventilatory threshold and V̇O_2_max, is recognized as an intensity zone associated with physiological markers that delineates the exercise intensity domains, such as critical power or critical speed (Souza et al. 2016; Jones et al. 2010; Lansley et al. 2011). Consequently, the inherent variability of this parameter may influence the specific intensity domain in which the exercise is performed, thereby altering the ${\rm{\dot V}}{{\rm{O}}_{{\rm{2}}}}$ kinetics response.

Another comparative study involving well-trained ultra-distance trail runners examined ${\rm{\dot V}}{{\rm{O}}_{{\rm{2}}}}$ kinetics during level and incline running (Willis et al. 2019), although it presented several limitations. Supposedly within the moderate domain, the authors arbitrarily selected the speeds and slopes (15 km·h^−1^ at 0% and 10 km·h^−1^ at 12%) for comparison, which resulted in greater ${\rm{\dot V}}{{\rm{O}}_{{\rm{2}}}}$ amplitude (41.3 ± 6.1 vs. 51.9 ± 6.9 ml·kg^−1^·min^−1^) and consequently greater time to reach ${\rm{\dot V}}{{\rm{O}}_{{\rm{2}}}}$ steady state (87.9 ± 31.6 vs. 108.1 ± 18.8 s) during uphill running. Nevertheless, the speed of phase II kinetics did not differ, as indicated by the primary time constant (10.2 ± 5.6 vs. 11.5 ± 6.4 s). Although this is an interesting finding in well-trained trail runners, caution is warranted, since the modelling of ${\rm{\dot V}}{{\rm{O}}_{{\rm{2}}}}$ kinetics was based on a single 5-min moderate-intensity transition, which is susceptible to the influence of outlying breaths (Whipp et al. 2005), in addition to aforementioned limitations.

A fundamental limitation in previous comparative studies resides in the difficulty of equating physiological demand across biomechanically distinct conditions. When comparisons rely on absolute workload matching (e.g., identical speed and slope across conditions for ${\rm{\dot V}}{{\rm{O}}_{{\rm{2}}}}$ kinetics assessment), metabolic demand differs substantially due to disparate mechanical efficiencies. Conversely, normalizing intensity via single-test criteria (e.g., applying the LT_1_ derived from a flat-running test to both conditions) compromises the physiological relevance, as biomechanical specificity is overlooked. The present study addresses this methodological challenge by independently determining intensity domain boundaries for each running condition, thereby facilitating intensity-matched comparisons while preserving biomechanical specificity.

One possibility to overcome these limitations is to compare the ${\rm{\dot V}}{{\rm{O}}_{{\rm{2}}}}$ on-kinetics during speed- and slope-specific exercise intensity domains (De Lucas et al. 2021). In this scenario, it is of interest to investigate whether specific slope-related modifications affect ${\rm{\dot V}}{{\rm{O}}_{{\rm{2}}}}$ on-kinetics, even when conducted in the same exercise intensity domains. This is particularly relevant considering that during trail running and orienteering races, the uphill running ability plays a key role in performance (Zürcher et al. 2005). Thus, considering that uphill running increases ${\rm{\dot V}}{{\rm{O}}_{{\rm{2SC}}}}$ amplitude (Pringle et al. 2002), but not the phase II kinetics primary time constant (Pringle et al. 2002; Willis et al. 2019), it was hypothesized that the primary component of phase II ${\rm{\dot V}}{{\rm{O}}_{{\rm{2}}}}$ on-kinetics would remain unaltered when exercise is performed within the moderate- and severe-intensity domains, despite a higher ${\rm{\dot V}}{{\rm{O}}_{{\rm{2SC}}}}$ amplitude in the severe domain during uphill compared to level running. While laboratory tests do not fully replicate the complexities of running on rugged terrain, they enable the control of exercise conditions from a physiological perspective, providing a robust framework to test the study’s hypothesis. Therefore, this study aimed to compare ${\rm{\dot V}}{{\rm{O}}_{{\rm{2}}}}$ on-kinetics during moderate- and severe-intensity domains during level and uphill treadmill running in trail runners.

## Materials and methods

### Participants

Sample size was determined based on the availability of experienced endurance trail runners in the region where the study was conducted who met the predefined inclusion criteria. Recruitment was carried out through running clubs, competitions, and social media platforms to maximize reach within this specific population. Given the relatively small and specialized nature of this population, the sample size reflects a resource- and population-constrained approach, which is an accepted strategy when access to eligible participants is limited. Importantly, as recommended by Lakens (2022), the justification of sample size should clarify how the collected data can still provide meaningful information in relation to the study’s inferential goals. In this context, the present study focuses on generating estimates and identifying potentially meaningful effects within a well-defined athletic population. Although no formal a priori power analysis was performed, the sample size is expected to yield informative effect size estimates and confidence intervals, contributing to the accumulation of evidence in this area (Ditroilo et al. 2025). Furthermore, the recruitment strategy aimed to include as many eligible participants as possible, thereby maximizing the informational value of the dataset within practical constraints.

The participants were 13 endurance trail runners (9 males and 4 females) classified as “trained” (Tier 2) and “highly trained” (Tier 3) (McKay et al. 2021). All athletes were involved in on- and off-road endurance running races for at least 4 years, and trained an average of 29.3 ± 10.1 km·week^− 1^ of running, with a weekly frequency of 3.4 ± 0.5 sessions. Inclusion criteria required male and female participants to have completed a 10 km run in under 45 min and 50 min, respectively, within the previous 12 months. The occurrence of any lower-limb injury in the last 3 months was considered an exclusion criterion. Participants volunteered and gave written informed consent to participate in this study, which was approved by the institutional ethics committee for research on humans (reference number 5.121.903) and was performed in accordance with the Declaration of Helsinki. This study was registered under the number RBR-4hx89v9 in the Brazilian Registry of Clinical Trials (ReBEC – ensaiosclinicos.gov.br/).

### Experimental design

**Fig. 1:**
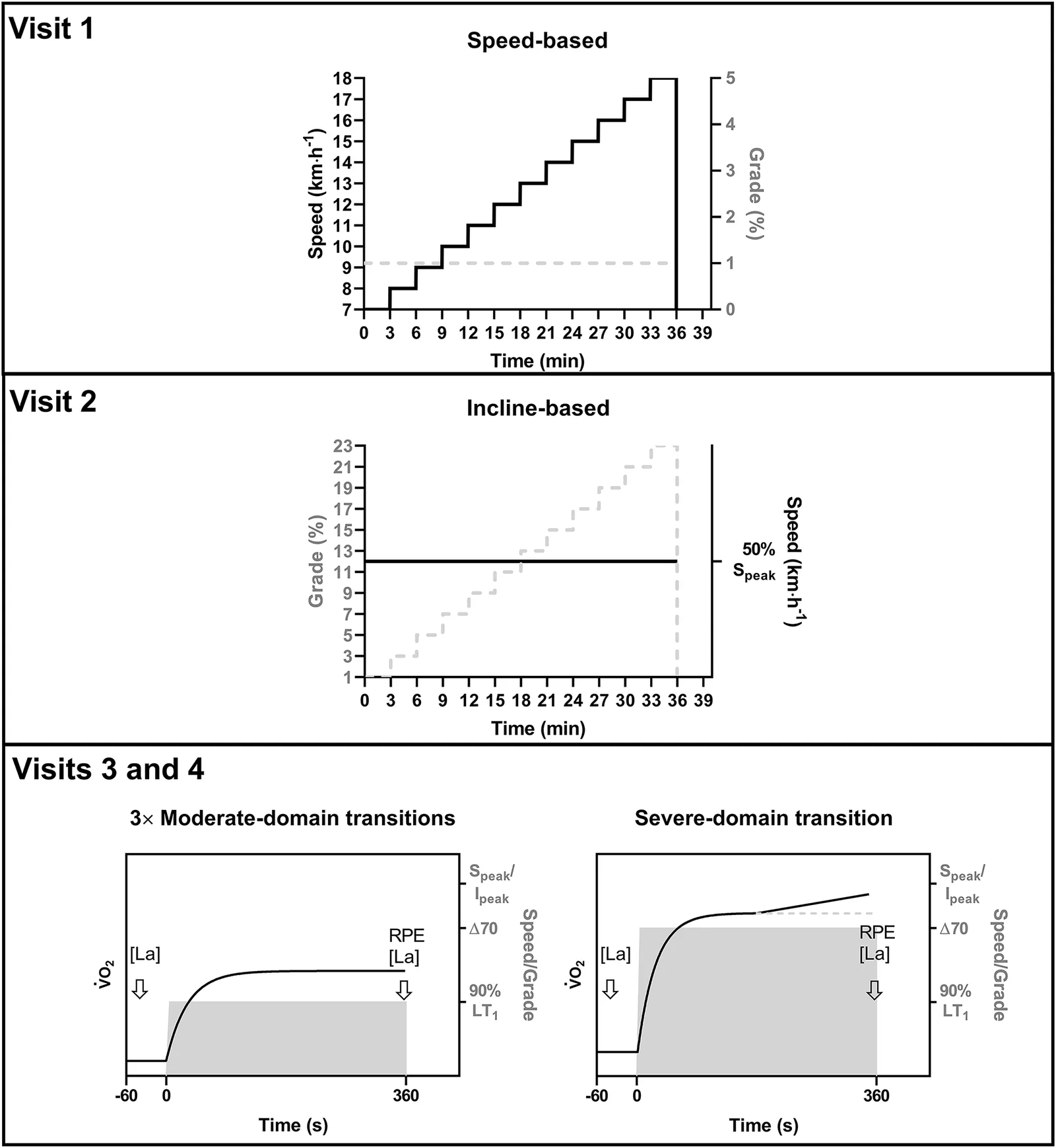
Schematic illustration of experimental design and sequence of tests. ${\rm{\dot V}}{{\rm{O}}_{{\rm{2}}}}$ oxygen uptake, *[La]* blood lactate concentration, *RPE* rating of perceived exertion, *S*_*peak*_ peak speed of speed-based incremental test, *I*_*peak*_ peak incline of inclined-based incremental test, *LT*_*1*_ first lactate threshold, *Δ70* intensity requiring 70 of the difference between the first lactate threshold and maximal oxygen uptake

In this experimental cross-sectional study, each participant visited the laboratory four times, with at least 72 h separating each visit, and all tests were completed within two weeks (Fig. 1). The participants first completed two separate speed-based and incline-based incremental tests to exhaustion to determine the LT_1_ and ${\rm{\dot V}}{{\rm{O}}_{{\rm{2max}}}}$ (De Lucas et al. 2021). During the subsequent visits, on two different and randomized days, participants completed a series of three 6-min square-wave transitions from rest to a workload (speed and slope combinations) calculated to require 90% of the ${\rm{\dot V}}{{\rm{O}}_{{\rm{2}}}}$ at LT_1_ (90% LT_1_ – moderate-intensity exercise) followed by one 6-min square-wave transition at 70% of the difference (Δ) between the ${\rm{\dot V}}{{\rm{O}}_{{\rm{2}}}}$ at LT_1_ and ${\rm{\dot V}}{{\rm{O}}_{{\rm{2max}}}}$ (Δ70 – severe-intensity exercise).

All the tests were conducted in a laboratory with controlled environmental conditions (air temperature at 20–22 °C and relative humidity at 60–70%). The protocols were performed on a motor-driven treadmill (Super ATL, Inbrasport, Porto Alegre, Brazil). Each participant was tested at the same time of day (± 1 h) to minimize the effects of diurnal biological variation. The participants refrained from high-intensity and/or long-distance training sessions for at least 48 h prior to testing. In addition, they were instructed to replicate their diet as closely as possible before each test.

### Incremental tests

The first incremental test was performed with the gradient set at 1% (i.e. Level), with an initial speed of 7 km·h^− 1^. Every 3 min the speed was increased by 1 km·h^− 1^, until volitional exhaustion (De Lucas et al. 2021). After at least 48 h, the incline-based (i.e. Uphill) incremental test was performed at a fixed speed (50% of the peak speed of the speed-based test) in which the incline started at 3%, and was increased by 2% every 3 min (De Lucas et al. 2021).

Breath-by-breath ${\rm{\dot V}}{{\rm{O}}_{{\rm{2}}}}$ data were continuously measured using an automated open-circuit gas analysis system (Quark CPET, Cosmed, Rome, Italy). Before each test, the O_2_ and CO_2_ analysis systems were calibrated using ambient air and a gas of known O_2_ (16%) and CO_2_ (5%) concentration according to the manufacturer’s instructions, while the turbine flow meter was calibrated using a 3-liter syringe (Cosmed, Rome, Italy). The ${\rm{\dot V}}{{\rm{O}}_{{\rm{2max}}}}$ was taken as the highest 15 s mean value achieved (Robergs et al. 2010). Heart rate was recorded (Polar H9, Kempele, Finland) and maximal heart rate (HRmax) was taken as the highest value attained. The highest speed and incline attained in the incremental tests were considered the respective S_peak_ and I_peak_. At the end of each stage, capillary blood samples (25 µl) were collected from the earlobe of each participant and blood lactate concentration ([La]) was measured using an electrochemical analyzer (YSI 2700 STAT, Yellow Springs, Ohio, USA). The LT_1_ was defined by the lowest [La]/workload ratio (lactate minimum equivalent – LME) as proposed by Dickhuth et al. (1999). This method has been demonstrated to be reliable for establishing physiological equivalence, specifically comparable ${\rm{\dot V}}{{\rm{O}}_{{\rm{2}}}}$, between speed-based and incline-based incremental tests at LT_1_ in endurance trail runners (de Lucas et al. 2021).

### Square-wave tests

During the same session, participants completed three moderate-exercise transitions at 90% of the incline-specific LT_1_ followed by one severe-exercise transition at the incline-specific Δ70, separated by 6 min of passive rest. Each 6-min transition was preceded by 2 min of passive rest on the treadmill. The [La] was determined immediately before and after the last moderate-exercise bouts and the severe-exercise bout to calculate the difference between pre- and post-exercise values (i.e., ∆[La]). In all conditions, ${\rm{\dot V}}{{\rm{O}}_{{\rm{2}}}}$ and HR were recorded throughout the sessions. The rating of perceived exertion (RPE) was asked to the participant at the end of each exercise bout using the Borg 6–20 scale (Borg 1982). Finally, a sagittal view of the participants was recorded (iPhone XR, Apple, USA) in well illuminated environment, using 4 K resolution and recording 60 frames per second, during 20 s in the second minute of the moderate and severe domain (from 120 to 140 s) to determine SF. Step length was then derived from the SF and the corresponding speed of the treadmill. The camera was positioned 0.5 m from the treadmill. Step frequency was manually counted frame-by-frame using KINOVEA software (v. 0.7.10) during the 20-second recording period by two independent observers. This has been established as a highly reliable method for step frequency determination (de Oliveira et al. 2019).

### ${\rm{\dot V}}{{\rm{O}}_{{\rm{2}}}}$ on-kinetics

Pulmonary ${\rm{\dot V}}{{\rm{O}}_{{\rm{2}}}}$ data from each test were initially examined to exclude occasional errant breaths caused by coughing, swallowing, or sighing, which were not considered reflective of the underlying kinetics; i.e., only values greater than three standard deviations (SD) from the local mean were omitted. The breath-by-breath data of only moderate-exercise were first linearly interpolated to provide the second-by-second values. Subsequently, for each individual, identical repetitions of each intensity were time aligned to the start of exercise and ensemble-averaged. The first 20 s of data after the onset of exercise (i.e., the phase I or cardio-dynamic response) were deleted, and a non-linear least-squares algorithm was used to fit the data thereafter. A single-exponential model was used to analyze ${\rm{\dot V}}{{\rm{O}}_{{\rm{2}}}}$ responses to moderate exercise, as described in the Eq. (1):

1 $$\:\dot{V}{O}_{2}\left(t\right)=\dot{V}{O}_{2}b+Ap\times\:\left({1-e}^{\left(-t-\frac{{TD}_{t}}{{\tau\:}_{p}}\right)}\right)$$

where ${\rm{\dot V}}{{\rm{O}}_{{\rm{2}}}}$(t) represents the absolute ${\rm{\dot V}}{{\rm{O}}_{{\rm{2}}}}$ at a given time t; ${\rm{\dot V}}{{\rm{O}}_{{\rm{2}}}}$b represents the mean ${\rm{\dot V}}{{\rm{O}}_{{\rm{2}}}}$ during the final 60 s of the baseline period; and Ap, TD_t_, and τ_p_ represent the amplitude, time delay, and time constant, respectively, describing the phase II increase in ${\rm{\dot V}}{{\rm{O}}_{{\rm{2}}}}$ above baseline.

For the severe domain, an iterative process ensured that the sum of squared errors was minimized. Due to large inter-individual differences in the duration of the exponential region (Murgatroyd et al. 2011), the identification of the ${\rm{\dot V}}{{\rm{O}}_{{\rm{2SC}}}}$ during the severe-intensity exercise was performed individually. The fundamental ${\rm{\dot V}}{{\rm{O}}_{{\rm{2}}}}$ on-kinetics (Phase II) was modeled following the iterative method to identify the exponential region (Murgatroyd et al. 2011; Rossiter et al. 2002). The “end” of the fundamental phase was performed by fitting a window from the start of the fundamental phase (i.e., after 20 s) and iteratively lengthened it until the exponential model fit demonstrated a discernible and consistent departure from the measured ${\rm{\dot V}}{{\rm{O}}_{{\rm{2}}}}$ values, following criteria proposed in the literature (Murgatroyd et al. 2011; Rossiter et al. 2002). Thus, the fitting window was constrained to this time point, and a single exponential fitting was performed only on the fundamental phase to identify the kinetics parameters. The model was constrained to the ${\rm{\dot V}}{{\rm{O}}_{{\rm{2}}}}$ baseline to aid the identification of the key parameters according to Eq. 1.

The ${\rm{\dot V}}{{\rm{O}}_{{\rm{2SC}}}}$ was then calculated according to Eq. (2):

2 $$\:\dot{V}{O}_{2SC}=\dot{V}{O}_{2}end-(\dot{V}{O}_{2}b+\:Ap)$$

**Fig. 2:**
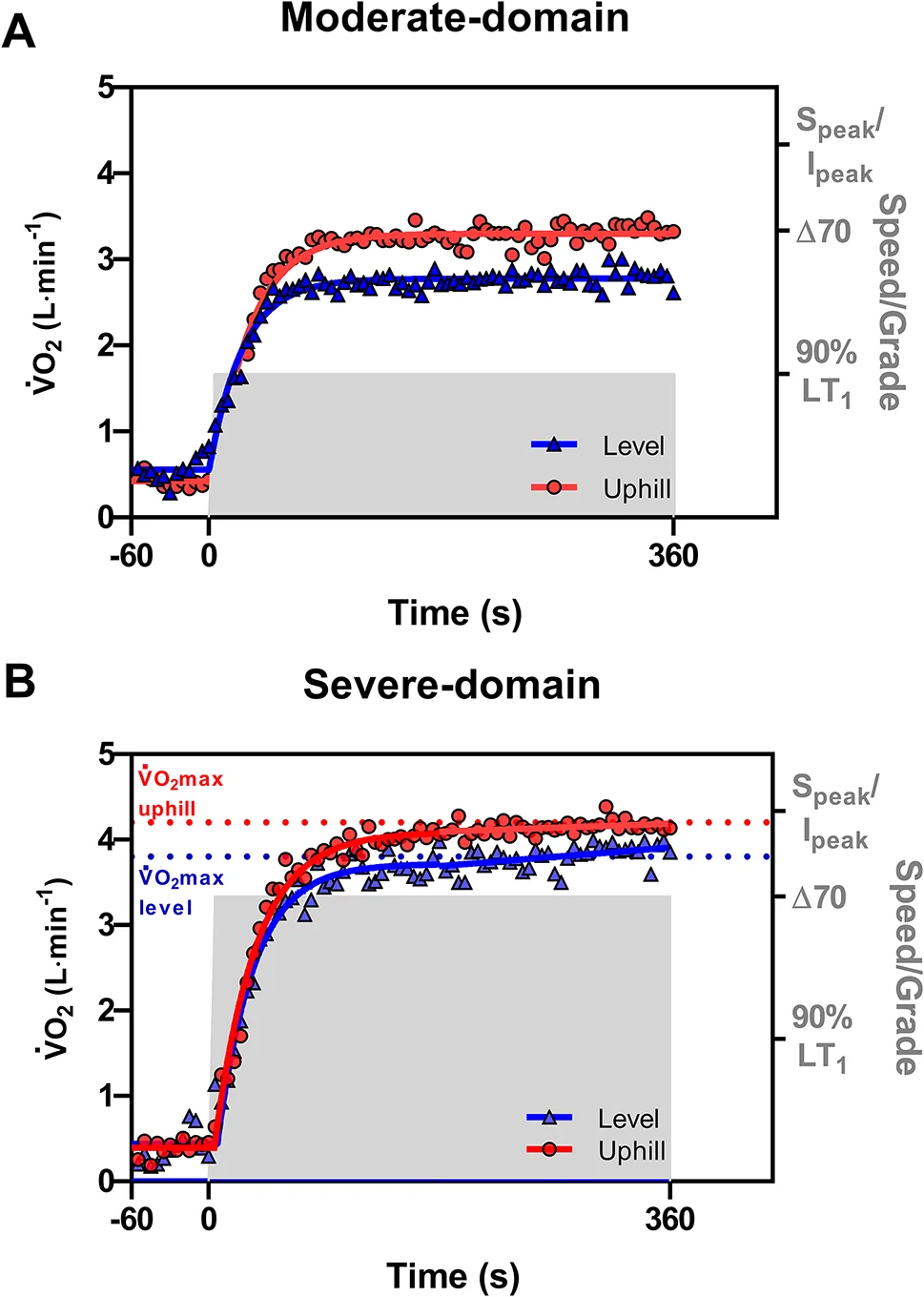
Oxygen uptake responses during moderate (**A**) and severe-intensity domains (**B**) for a representative subject. In panel **A**, horizontal dashed lines indicate ${\rm{\dot V}}{{\rm{O}}_{{\rm{2max}}}}$ values obtained during level (blue) and uphill (red) incremental tests. Data in the severe-intensity domain are based on a single transition, whereas in the moderate-intensity domain each ${\rm{\dot V}}{{\rm{O}}_{{\rm{2}}}}$ data point represents the time-aligned average of three transitions ${\rm{\dot V}}{{\rm{O}}_{{\rm{2}}}}$ oxygen uptake; ${\rm{\dot V}}{{\rm{O}}_{{\rm{2max}}}}$ maximal oxygen uptake, *S*_*peak*_ peak speed of speed-based incremental test, *I*_*peak*_ peak incline of inclined-based incremental test, *LT*_*1*_ first lactate threshold, *Δ70* intensity requiring 70 of the difference between the first lactate threshold and maximal oxygen uptake

Where ${\rm{\dot V}}{{\rm{O}}_{{\rm{2}}}}$end is the average V̇O_2_ value over the last 15 s of the 6-min running exercise, ${\rm{\dot V}}{{\rm{O}}_{{\rm{2}}}}$b represents the mean ${\rm{\dot V}}{{\rm{O}}_{{\rm{2}}}}$ in the final 60 s of the baseline period, and Ap represents the amplitude ${\rm{\dot V}}{{\rm{O}}_{{\rm{2}}}}$ above baseline. Time delay before the onset of V̇O_2SC_ (V̇O_2SC_ TD_t_) was considered the time at the “end” of the fundamental phase identified through the iterative process. The ${\rm{\dot V}}{{\rm{O}}_{{\rm{2}}}}$ responses to moderate and severe exercise and the respective kinetic fitting for a representative participant are illustrated in Fig. 2.

### Statistical analysis

Descriptive data are presented as means ± SD and statistical parameters as mean point estimates with 95% confidence intervals (CI). Paired-sample Student’s t-test were utilized to examine differences between level and uphill running. Two-way repeated measures ANOVA was performed to compare [La] responses between conditions. When significant differences were found, Bonferroni post hoc test were performed to identify the source of the differences. Standardized effect sizes were calculated using Cohen’s *d* to quantify the magnitude of differences between level and uphill conditions for ${\rm{\dot V}}{{\rm{O}}_{{\rm{2}}}}$ kinetics parameters. Cohen’s *d* was interpreted according to the following scale: trivial (< 0.2), small (0.2–0.6), moderate (0.6–1.2), large (1.2–2.0), very large (2.0–4.0), and extremely large (> 4.0) (Hopkins et al. 2009). We defined the trivial region as the equivalence region. If the entire 95% CI is contained within this region, the comparison is considered equivalent. If the 95% CI crosses the ± 0.2 boundaries, the effect is considered inconclusive. If the 95% CI lies entirely outside the trivial region, the comparison is considered non-equivalent (Ialongo 2017). All analyses were carried out using GraphPad Prism software, version 8.1.2 (GraphPad Software, La Jolla, CA, USA). The level of significance was set at *p* < 0.05.

## Results

Males were 36 ± 10 years old, 175 ± 6 cm tall and weighed 70 ± 7 kg, whereas females were 41 ± 5 years old, 164 ± 11 cm tall and weighed 61 ± 6 kg. During the incremental tests, participants’ ${\rm{\dot V}}{{\rm{O}}_{{\rm{2}}}}$peak values were not significantly different between conditions (Speed-based: 3.87 ± 0.80 vs. Incline-based: 3.95 ± 0.75 L·min^−1^; *p* = 0.340), defining it as a ${\rm{\dot V}}{{\rm{O}}_{{\rm{2max}}}}$. However, participants’ HRmax was lower during the incline-based compared to the speed-based incremental test (184 ± 13 vs. 180 ± 10 bpm; *p* = 0.031). The S_peak_ and I_peak_ were 16.3 ± 1.8 km·h^−1^ and 15.8 ± 0.9%, respectively. The ${\rm{\dot V}}{{\rm{O}}_{{\rm{2}}}}$ at LT_1_ was significantly higher during inclined-based (2.82 ± 0.94 L·min⁻¹) compared to speed-based incremental test (2.68 ± 0.56 L·min⁻¹; *p* = 0.013).

The ${\rm{\dot V}}{{\rm{O}}_{{\rm{2}}}}$ on-kinetics parameters for the moderate domain are presented in Table 1. The baseline ${\rm{\dot V}}{{\rm{O}}_{{\rm{2}}}}$, time delay, and the primary time constant were not significantly different between conditions (*p* > 0.05). However, the ${\rm{\dot V}}{{\rm{O}}_{{\rm{2}}}}$ primary amplitude was significantly higher during uphill running than during level running (*p* = 0.042).

**Table 1:** Oxygen uptake on-kinetics during moderate exercise (i.e. 90% LT_1_) at level and uphill running

Variable	Mean ± SD		Mean difference (95% CI)	*p*-value
	Level	Uphill		
Speed (km.h^− 1^)	9.9 ± 1.1	8.2 ± 0.9	NA	NA
Incline (%)	1.0 ± 0.0	7.1 ± 1.1	NA	NA
${\rm{\dot V}}{{\rm{O}}_{{\rm{2}}}}$b (L·min^−1^)	0.43 ± 0.07	0.43 ± 0.07	0.00 (− 0.06 to 0.06)	0.846
Ap (L·min^− 1^)	2.06 ± 0.54	2.33 ± 0.51*	0.27 (0.01–0.54)	0.042
TD_t_ (s)	12.5 ± 4.0	11.7 ± 4.5	− 1.8 (− 5.4 to 1.9)	0.382
τ_p_ (s)	16.0 ± 5.7	18.7 ± 5.9	2.7 (1.3–6.7)	0.162

*NA* non-applicable; ${\rm{\dot V}}{{\rm{O}}_{{\rm{2}}}}$*b* baseline oxygen uptake; *Ap* primary amplitude, *TD*_*t*_ time delay; *τ*_*p*_ primary time constant

*Significant difference from level running (*p* < 0.05)

**Fig. 3:**
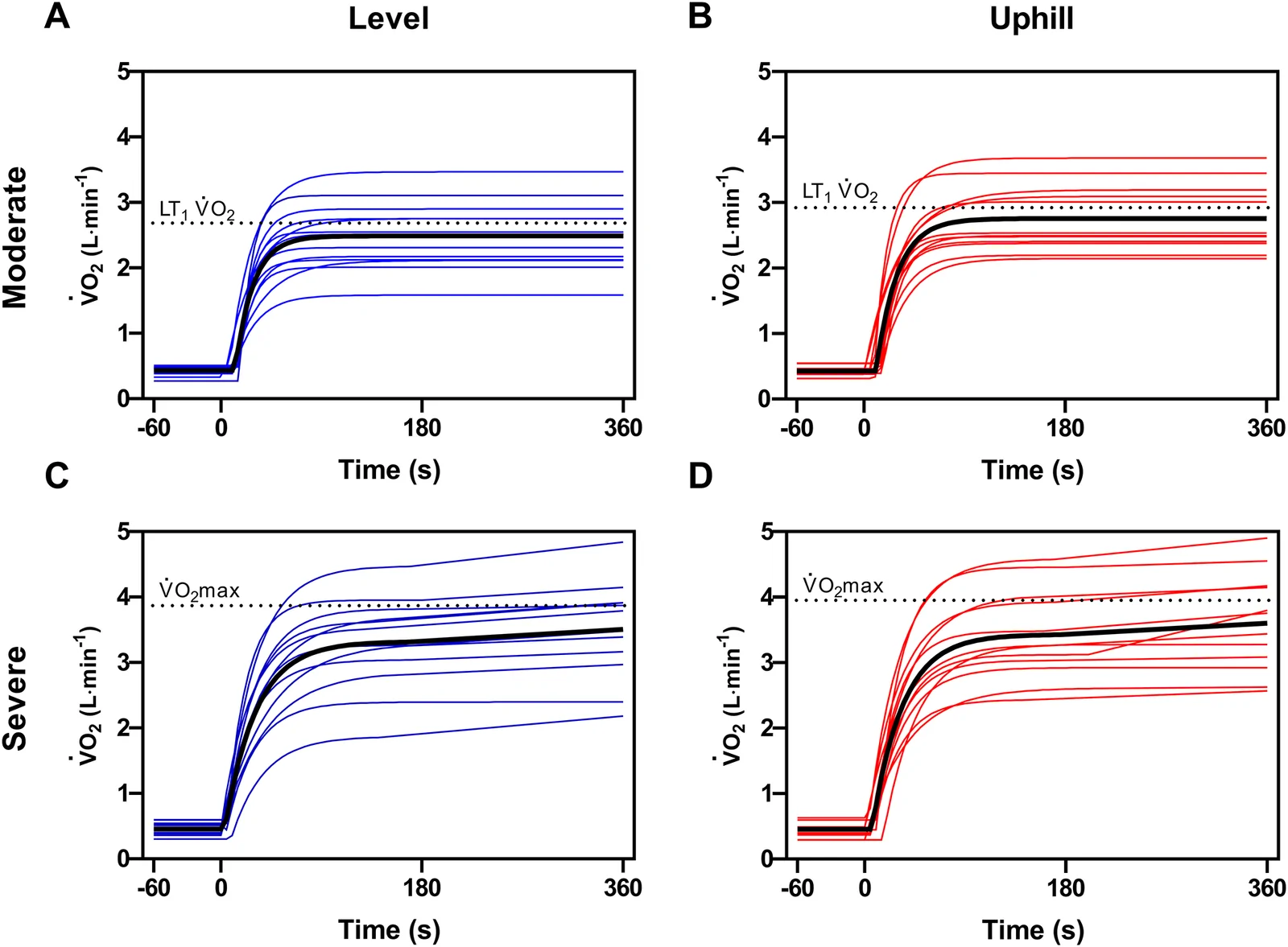
Oxygen uptake kinetics during moderate- and severe-intensity running in level and uphill conditions. Panels **A** and **B** show moderate-intensity responses for level (blue) and uphill (red) running, respectively, while panels C and D present severe-intensity responses for level (blue) and uphill (red) running. Thin lines represent individual responses, and thick black lines indicate the sample mean. In the moderate-intensity domain, ${\rm{\dot V}}{{\rm{O}}_{{\rm{2}}}}$ responses were obtained from three time-aligned transitions (ensemble-averaged), whereas in the severe-intensity domain responses are based on a single transition. Horizontal dashed lines indicate the sample mean ${\rm{\dot V}}{{\rm{O}}_{{\rm{2}}}}$ at the first lactate threshold (LT_1_) in panels A and B and sample mean ${\rm{\dot V}}{{\rm{O}}_{{\rm{2max}}}}$ in panels **C** and **D**. Time zero represents the onset of exercise

No significant differences were observed in baseline ${\rm{\dot V}}{{\rm{O}}_{{\rm{2}}}}$, primary ${\rm{\dot V}}{{\rm{O}}_{{\rm{2}}}}$ amplitude, time delay, time constant, ${\rm{\dot V}}{{\rm{O}}_{{\rm{2SC}}}}$ amplitude, or time delay between conditions (Table 2). Peak ${\rm{\dot V}}{{\rm{O}}_{{\rm{2}}}}$ was higher during the uphill (3.86 ± 0.81 L∙min^−1^) compared to the level (3.65 ± 0.78 L∙min^−1^; *p* = 0.037) protocol. However, there were no significant difference between the peak ${\rm{\dot V}}{{\rm{O}}_{{\rm{2}}}}$ during level (*p* = 0.117) and uphill (*p* = 0.273) conditions when compared with the ${\rm{\dot V}}{{\rm{O}}_{{\rm{2max}}}}$ from their respective incremental tests. The sample mean and individual ${\rm{\dot V}}{{\rm{O}}_{{\rm{2}}}}$ kinetics at moderate and severe exercise are presented in Fig. 3.

**Table 2:** Oxygen uptake on-kinetics during severe exercise (i.e. Δ70%) at level and uphill running

Variable	Mean ± SD		Mean difference (95% CI)	*p*-value
	Level	Uphill		
Speed (km.h^− 1^)	14.7 ± 1.7	8.2 ± 0.9	NA	NA
Incline (%)	1.0 ± 0.0	13.1 ± 0.7	NA	NA
${\rm{\dot V}}{{\rm{O}}_{{\rm{2}}}}$b (L·min^−1^)	0.46 ± 0.10	0.46 ± 0.09	0.01 (− 0.08 to 0.09)	0.969
Ap (L·min^− 1^)	2.86 ± 0.69	2.98 ± 0.66	0.11 (− 0.16 to 0.38)	0.384
TD_t_ (s)	3.5 ± 3.4	6.7 ± 5.1	3.2 (− 0.3 to 6.7)	0.07
τ_p_ (s)	28.0 ± 7.1	27.1 ± 5.0	− 0.9 (− 4.6 to 2.7)	0.584
${\rm{\dot V}}{{\rm{O}}_{{\rm{2SC}}}}$ Ap (L·min^−1^)	0.21 ± 0.10	0.21 ± 0.19	0.00 (− 0.15 to 0.15)	0.961
${\rm{\dot V}}{{\rm{O}}_{{\rm{2SC}}}}$ TD_t_ (s)	169.2 ± 45.6	173.3 ± 27.7	4.2 (− 20.4 to 28.8)	0.716

*NA* non-applicable; ${\rm{\dot V}}{{\rm{O}}_{{\rm{2}}}}$*b* baseline oxygen uptake; *Ap* primary amplitude, *TD*_*t*_ time delay; *τ*_*p*_ primary time constant; ${\rm{\dot V}}{{\rm{O}}_{{\rm{2SC}}}}$ *Ap* slow component amplitude of oxygen uptake; ${\rm{\dot V}}{{\rm{O}}_{{\rm{2SC}}}}$ *TD*_*t*_ time delay before the onset of oxygen uptake slow component

**Fig. 4:**
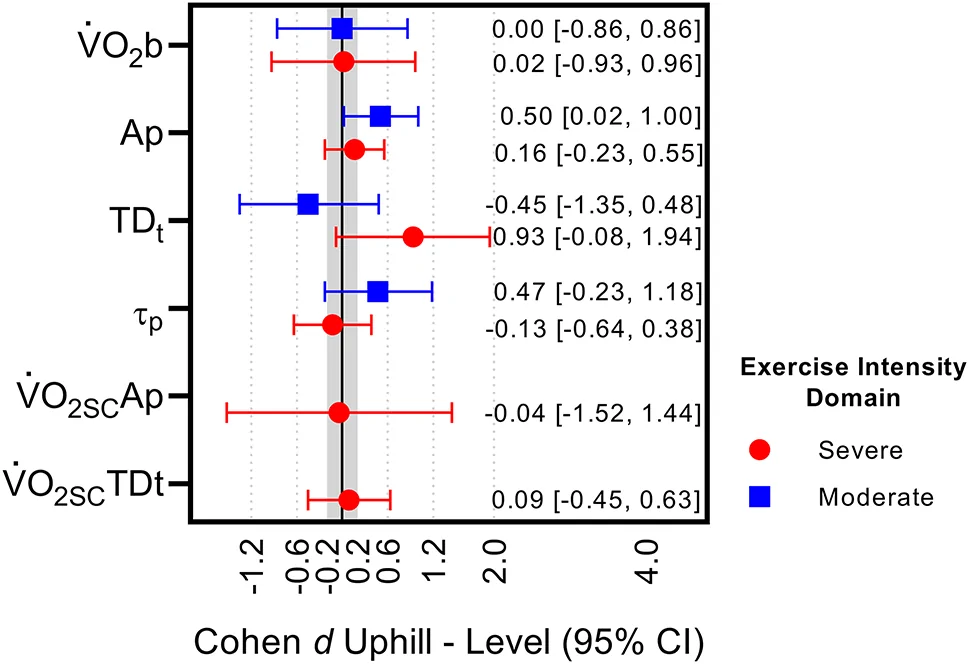
Standardized effect sizes (Cohen’s *d*) for the comparison between uphill and level running across ${\rm{\dot V}}{{\rm{O}}_{{\rm{2}}}}$ kinetics parameters in moderate and severe intensity domains. Data are presented as mean standardized effect sizes with [95% confidence intervals]. The vertical dashed lines present the magnitude thresholds of trivial (gray shaded region; |0.20|), small (|0.20| to |0.60|), moderate (|0.60| to |1.20|), large (|1.20| to |2.00|), and very large (|2.00| to |4.00|) effects

Standardized differences (i.e., Cohen’s *d*) between level and uphill conditions for ${\rm{\dot V}}{{\rm{O}}_{{\rm{2}}}}$ kinetic parameters during moderate- and severe-intensity exercise are presented in Fig. 4. The magnitudes of the differences were “trivial” for the majority of comparisons, except for TD_t_ in both moderate (*d* = − 0.45, “small”) and severe (*d* = 0.93, “moderate”) exercise, and τ_p_ during moderate exercise (*d* = 0.47, “small”). Nevertheless, for all effects, the 95% confidence intervals crossed the − 0.2 and/or 0.2 boundaries of a “trivial” effect, indicating that no effect could be considered equivalent within this predefined equivalence region.

With regard to [La] in the moderate domain, a significant interaction between time and condition (F_(1, 40)_ = 4.443, *p* = 0.041) effect was found. There were no differences in pre-exercise [La] between conditions (*p* = 0.868), while post-exercise [La] was higher in uphill compared to level running (*p* = 0.003). [La] increased from pre- to post-exercise during uphill (*p* = 0.026) but not during level running (*p* = 0.999). For the [La] in the severe domain, significant main effects for time (F_(1, 44)_ = 215.1, *p* < 0.001), and condition (F_(1, 44)_ = 6.125, *p* = 0.017) were observed, with no significant interaction (F_(1, 44)_ = 1.291, *p* = 0.262). [La] increased from pre- to post-exercise in both conditions (*p* < 0.01), with no differences in the pre- (*p* = 0.923) or post-exercise (*p* = 0.082) values between conditions. The ∆[La] at moderate intensity was higher during the uphill compared to level running (*p* = 0.048), but not in the severe domain (*p* = 0.123) (Table 3).

**Table 3:** Blood lactate concentration during moderate and severe exercise at level and uphill running

Variable	Level	Uphill	Level	Uphill
	Moderate		Severe	
Pre-exercise (mmol·L^− 1^)	1.38 ± 0.33	1.23 ± 0.30	1.15 ± 0.36	1.57 ± 0.54
Post-exercise (mmol·L^− 1^)	1.24 ± 0.54	1.96 ± 0.65*^$^	5.41 ± 1.27*	6.55 ± 1.65*
∆[La] (mmol·L^− 1^)	0.11 ± 0.54	0.58 ± 0.64^$^	4.26 ± 1.13	4.98 ± 1.69

Data presented as mean ± SD. *∆[La]* difference between pre-to-post exercise in blood lactate concentration

* Significant difference from pre-exercise in the same condition (*p* < 0.05)

^$^Significant difference from level running in the same moment (*p* < 0.05)

There were no differences in final HR for either moderate (Level: 137 ± 10 vs. Uphill: 146 ± 11 bpm, *p* = 0.078) or severe intensities (Level: 171 ± 8 vs. Uphill: 175 ± 11 bpm, *p* = 0.071). Ratings of perceived exertion (RPE) were not different for the moderate (Level: 10 ± 2 vs. Uphill: 12 ± 2, *p* = 0.087) or severe domains (Level: 16 ± 2 vs. Uphill: 17 ± 2, *p* = 0.154).

During the moderate domain, a greater step frequency (178 ± 7 vs. 175 ± 7 steps·min^− 1^; *p* = 0.029) and step length (0.92 ± 0.11 vs. 0.78 ± 0.09 m; *p* < 0.01) were observed during level compared to uphill running. Higher values were also observed for the severe domain for step frequency (189 ± 9 vs. 177 ± 8 steps·min^− 1^; *p* < 0.01) and step length (1.30 ± 0.17 vs. 0.77 ± 0.09 m, *p* < 0.01) during level compared to uphill running.

## Discussion

To investigate the influence of uphill running on ${\rm{\dot V}}{{\rm{O}}_{{\rm{2}}}}$ on-kinetics, an intensity-matched comparison based on slope-specific thresholds was conducted by accurately determining the exercise intensity domains derived from two specific incremental tests, which increases the confidence in the current findings. The main finding of the present study was the similarity in the time parameters (i.e., TD_t_ and τ_p_) of ${\rm{\dot V}}{{\rm{O}}_{{\rm{2}}}}$ on-kinetics between level and uphill treadmill running in trail runners, during moderate- and severe-intensity domains. These results support the primary hypothesis that the fundamental components of ${\rm{\dot V}}{{\rm{O}}_{{\rm{2}}}}$ on-kinetics would remain unaltered during moderate and severe intensities when normalized between the running conditions. However, the results did not support the hypothesis that uphill running would induce a higher ${\rm{\dot V}}{{\rm{O}}_{{\rm{2SC}}}}$ amplitude at the severe intensity compared to level-running. Thus, when exercise intensities are individually normalized within the same domain, the augmented ratio of concentric to eccentric muscle activation does not affect ${\rm{\dot V}}{{\rm{O}}_{{\rm{2}}}}$ on-kinetics in the moderate and severe domains.

Few studies have compared ${\rm{\dot V}}{{\rm{O}}_{{\rm{2}}}}$ kinetics between level and uphill running. One potential difficulty is normalizing intensities while maintaining the ‘real life’ mechanical demand of uphill running. For instance, Pringle et al. (2002) compared by using intensity parameters derived from level (0%) and fixed slope (10%) incremental tests, while Willis et al. (2019) chose arbitrary speeds and slopes to compare conditions. In the present study, uphill running increased primary ${\rm{\dot V}}{{\rm{O}}_{{\rm{2}}}}$ amplitude and [La] in comparison with level running during moderate exercise. In contrast, Pringle et al. (2002) observed similar values for ∆[La] between level and uphill running during moderate-intensity exercise (80% of ventilatory threshold) based on specific level and incline incremental tests. However, the incline incremental protocol employed in their study utilized a fixed 10% slope with speed-based increments, which differs from the incremental protocol used in the present study. Thus, the present study is the first to perform a constant work rate test based on parameters derived from incremental tests conducted with both fixed speed and slope increments.

However, the main explanation for the differences in the findings of the present study may be related to the determination of LT_1_. Indeed, ${\rm{\dot V}}{{\rm{O}}_{{\rm{2}}}}$ at LT_1_ was higher in the incline-based incremental test compared to speed-based incremental test. Considering that the constant workload exercise intensity was individually normalized according to the LT_1_ obtained in each incremental test, it is likely that the greater ${\rm{\dot V}}{{\rm{O}}_{{\rm{2}}}}$ amplitude and [La] observed during uphill running resulted from the higher metabolic demand associated with LT_1_ in the incline-based than in the speed-based incremental test. However, the choice of the method used to determine LT_1_ in the present study was based on a previous study that did not observe differences in ${\rm{\dot V}}{{\rm{O}}_{{\rm{2}}}}$ at LT_1_ using the same approach (de Lucas et al. 2021). Given that the method used considers only the relationship between [La] and workload during the incremental test to determine LT_1_, the intensity at which the moderate exercise was performed did not depend on prior ${\rm{\dot V}}{{\rm{O}}_{{\rm{2}}}}$ analysis. Thus, the overall data analysis was performed after all experimental sessions, and the comparisons that detected this difference were conducted only after all procedures had been completed. Indeed, a recent study demonstrated that the LME method for determining LT_1_ was the only model that showed a significant difference in V̇O₂ between speed-based and incline-based incremental tests (Ventura et al. 2026). Therefore, the differences observed in ${\rm{\dot V}}{{\rm{O}}_{{\rm{2}}}}$ and [La] between level and uphill running during moderate exercise are more likely related to a methodological issue rather than a physiological one.

The current results showed no differences in ${\rm{\dot V}}{{\rm{O}}_{{\rm{2SC}}}}$ amplitude within severe intensity domain between running conditions, refuting the proposed hypothesis. The increased ratio of concentric to eccentric contraction in uphill compared to level running leads to reduced metabolic-to-work efficiency, primary due to reduced elastic energy storage and higher motor unit recruitment (Minetti et al. 1994; Aura and Komi 1986). In addition, greater muscle mass recruitment is observed during uphill running, eliciting greater ${\rm{\dot V}}{{\rm{O}}_{{\rm{2}}}}$ responses (De Lucas et al. 2025). Together, these factors could be expected to trigger a larger ${\rm{\dot V}}{{\rm{O}}_{{\rm{2SC}}}}$ amplitude in uphill running compared to level running, as previously reported (Pringle et al. 2002). Pringle et al. (2002) found an increase in ${\rm{\dot V}}{{\rm{O}}_{{\rm{2SC}}}}$ during uphill (10%) compared to level (0%) running within the heavy intensity domain, determined using 50% of the difference between the first ventilatory threshold and ${\rm{\dot V}}{{\rm{O}}_{{\rm{2}}}}$max (50% Δ) in both conditions. The exercise at 50%Δ has been associated with the intensity that delineates the transition between the heavy and severe domains (i.e., critical power/critical speed) (Souza et al. 2016; Jones et al. 2010). Considering that ${\rm{\dot V}}{{\rm{O}}_{{\rm{2}}}}$ kinetics respond differently in heavy and severe exercise-intensity domains (Poole and Jones 2012), and the variability with which this physiological marker may occur (Souza et al. 2016; Jones et al. 2010; Lansley et al. 2011), it is possible that the conditions compared by Pringle et al. (2002) may have occurred in distinct physiological domains.

These conflicting results regarding ${\rm{\dot V}}{{\rm{O}}_{{\rm{2SC}}}}$ responses between level and uphill running may be related to exercise intensity normalization, which remains a major challenge when comparing physiological responses between different running conditions. In this regard, the specific protocol for uphill running utilized in the present study allows for a more precise replication of uphill demands (De Lucas et al. 2021). Therefore, it is possible that the divergences between the current and previous studies (Pringle et al. 2002) can be attributed to a lack of standardization of intensities between level and uphill conditions, as well as to the specificity of running speeds during uphill exercise. In addition, individuals with lower aerobic fitness and with little exposure to uphill running may present different ${\rm{\dot V}}{{\rm{O}}_{{\rm{2SC}}}}$ responses (Reis et al. 2007; Scheer et al. 2018).

When running is performed at the same speed, increasing the incline raises step frequency and decreases step length (Balducci et al. 2016; Padulo et al. 2013). This increase in step frequency has been indicated as a factor contributing to higher metabolic demand during incline running compared with level running (Padulo et al. 2013). The results of the present study, together with those reported by Pringle et al. (2002), reinforce this perspective, specifically when running was performed at the same relative intensity specific to each condition, step frequency was lower during the uphill protocol compared with level running. Therefore, using testing protocols specific to different running conditions (incline or level) is essential for accurately determining exercise intensity during a training session, as each condition implies relevant physiological and biomechanical adaptations.

Despite the interesting findings, this study is not without limitations. Most critically, the severe-intensity domain analysis is based on single transitions per condition per participant. Established methodology for ${\rm{\dot V}}{{\rm{O}}_{{\rm{2}}}}$ kinetics characterization recommends at least duplicate transitions to assess within-participant reliability (Buchheit et al. 2012; Clark and Draper 2019). While the comparability of peak ${\rm{\dot V}}{{\rm{O}}_{{\rm{2}}}}$ to incremental test values demonstrates that participants exercised within the intended severe-intensity domain, this does not constitute evidence of test-retest reliability for kinetic parameters. Typical within-participant coefficients of variation for τp during repeated severe-intensity transitions range from 10 to 20% (Murgatroyd et al. 2011). Consequently, the observed difference in τ_p_ between conditions (0.9 s) may fall within measurement error bounds, and future studies should be conducted with two or more transitions in the severe domain to confirm the reliability of the data from the present study.

Additionally, a limitation of the present study is the relatively small sample size. However, this reflects the specific and hard-to-access population investigated, namely experienced endurance trail runners meeting strict inclusion criteria. Studies involving highly trained or specialized athletic populations commonly face such constraints due to limited availability and recruitment feasibility (Lakens 2022). Despite this, the consistency of the observed responses and the use of repeated measures strengthen the internal validity of the findings. Importantly, the present results should be interpreted as hypothesis-generating, providing a foundation for future studies with larger samples to confirm and extend these findings (Ditroilo et al. 2025). Furthermore, the findings of the present study should be interpreted with caution when extrapolated to real-world conditions of this sport. The tests were conducted under controlled laboratory settings to isolate the effects of different running inclinations, which was necessary to test the study hypothesis. However, such conditions may not fully capture the physiological and biomechanical complexities inherent to real trail running environments. Finally, the cohort consisted exclusively of trained trail runners; the findings may not generalize to recreational runners or to populations with limited experience on inclined surfaces.

It is important to highlight that trail runners possess specific adaptations due to the nature of their sport (Pastor et al. 2023). Mountain and ultra-trail running involve substantial elevation changes, which demand both concentric (uphill) and eccentric (downhill) efforts. Therefore, a specific test that accounts for these particularities of trail running is essential for evaluating physiological and biomechanical parameters and for guiding training prescription. The findings of the present study reinforce the accuracy of slope-specific incremental test for prescribing exercise intensity based on the same physiological parameters used in conventional protocols, as metabolic responses were similar across both intensity domains. Although a higher primary ${\rm{\dot V}}{{\rm{O}}_{{\rm{2}}}}$ amplitude and a greater Δ[La] were observed during uphill compared to level exercise at the same relative intensity (90% LT_1_), both conditions were performed within the moderate-intensity domain. This indicates that the higher metabolic demand found during uphill running in the present study can be attributed to the predominance of concentric muscle action in this condition compared to level running, which limits the use of elastic energy stored in the muscle and requires greater mechanical work to overcome the gradient (Snyder and Farley 2011).

## Conclusion

In conclusion, the slope-specific incremental test effectively defines exercise-intensity domains in trail runners, yielding similar ${\rm{\dot V}}{{\rm{O}}_{{\rm{2}}}}$ on-kinetics between level and uphill running. Despite the higher primary ${\rm{\dot V}}{{\rm{O}}_{{\rm{2}}}}$ amplitude and Δ[La] during uphill running, these responses remained consistent with the moderate-intensity domain. Finally, ${\rm{\dot V}}{{\rm{O}}_{{\rm{2SC}}}}$ was similar during exercise within the severe intensity domain in both conditions, supporting the validity of incline-based testing for physiological normalization.

## Abbreviations

[La]: Blood lactate concentration
∆[La]: Difference between pre-to-post exercise in blood lactate concentration
Δ70: Intensity requiring 70% of the difference between the first lactate threshold and maximal oxygen uptake
95% CI: Confidence interval of 95%
Ap: Primary amplitude
I_peak_: Peak incline of inclined-based incremental test
LT_1_: First lactate threshold
RPE: Rating of perceived exertion
SF: Step frequency
SL: Step length
S_peak_: Peak speed of speed-based incremental test
τ_p_: Primary time constant
TD_t_: Time delay
$${\rm{\dot V}}{{\rm{O}}_{\rm{2}}}$$: Oxygen uptake
$\dot V{O_2}b$: Baseline oxygen uptake
${\rm{\dot V}}{{\rm{O}}_{{\rm{2max}}}}$: Maximal oxygen uptake
${\rm{\dot V}}{{\rm{O}}_{{\rm{2SC}}}}$: Slow component of oxygen uptake
$\dot V{O_2}_{SC}Ap$: Slow component amplitude of oxygen uptake
$\dot V{O_2}_{SC}T{D_t}$: Time delay before the onset of oxygen uptake slow component
